# CS2164 and Venetoclax Show Synergistic Antitumoral Activities in High Grade B-Cell Lymphomas With *MYC* and *BCL2* Rearrangements

**DOI:** 10.3389/fonc.2021.618908

**Published:** 2021-03-10

**Authors:** Delin Yuan, Genhong Li, Lian Yu, Yuelong Jiang, Yuanfei Shi, Qiulin Chen, Xiaomei Ma, Lan V. Pham, Ken H. Young, Manman Deng, Zhihong Fang, Bing Xu

**Affiliations:** ^1^ Department of Hematology, The First Affiliated Hospital of Xiamen University and Institute of Hematology, School of Medicine, Xiamen University, Xiamen, China; ^2^ Department of Hematology, Key Laboratory of Xiamen for Diagnosis and Treatment of Hematological Malignancy, Xiamen, China; ^3^ Department of Hematology and Rheumatology, Longyan First Hospital Affiliated to Fujian Medical University, Longyan, China; ^4^ Biology, Tumor Dependency, Phamacyclics, Abbvie Company, San Francisco, CA, United States; ^5^ Division of Hematopathology, Department of Pathology, Duke University Medical Center, Duke University, Durham, NC, United States

**Keywords:** HGBL-DHL, MYC, BCL2, CS2164, venetoclax, synergy/synergism, combination therapy, PI3K signaling

## Abstract

High-grade B-cell lymphoma with concurrent *MYC* and *BCL2* rearrangements (HGBL-DHL) is a rare, aggressive mature B-cell malignancy with a high likelihood of treatment failure following front-line immunochemotherapies. Patients with HGBL-DHL who develop a relapsed or refractory disease have little effective therapeutic strategies and show very poor clinical outcomes, thus calling for development of novel therapies for this specific patient population. In this study, we investigated the preclinical anti-lymphoma efficacies and potential mechanism of action of a novel treatment approach, combining the BCL2 inhibitor venetoclax with CS2164, a new orally active multitarget inhibitor, in HGBL-DHL models. This combination therapy exhibited a robust synergistic cytotoxicity against HGBL-DHL cells, evidenced by cooperatively inducing loss of cell viability and promoting cell apoptosis. Moreover, coadministration of CS2164 and venetoclax resulted in significant superior suppression of HGBL-DHL cell growth and remarkably abrogated tumor burden in a HGBL-DHL-xenografted mouse model. The synergistic lethality of CS2164 and venetoclax in HGBL-DHL cells was associated with induction of DNA damage and impairment of DNA repair ability. Of importance, the combined treatment almost abolished the expression of both BCL2 and MYC, two hallmark proteins of HGBL-DHL, and substantially blunted the activity of PI3K/AKT/mTOR signaling cascade. In addition, MCL1 and BCL-XL, two well-characterized contributors for venetoclax resistance, were significantly lessened in the presence of CS2164 and venetoclax, thus leading to the accumulation of proapoptotic proteins BAX and PUMA and then initiating the intrinsic apoptosis pathway. Taken together, these findings suggest that the regimen of CS2164 and venetoclax is highly effective to eliminate HGBL-DHL cells in the preclinical setting, warranting further clinical investigations of this regimen for the treatment of unfavorable HGBL-DHL patients.

## Introduction

High grade B-cell lymphoma with MYC and BCL2 rearrangements (HGBL-DHL), formerly termed double-hit lymphoma (DHL) which constitutes 5–10% of histologically diagnosed diffuse large B-cell lymphoma (DLBCL), is currently classified as a unique and separate entity (1, 2). Despite recently significant advances in other lymphomas, patients with HGBL-DHL remain highly refractory and resistant to standard of care with dismal clinical outcomes (3–5). Even administration of stem cell transplantation that serves as a potential curative option for HGBL-DHL cases, the prognosis of this patient population still has not been largely improved with median overall survival inferior to 2 years (6). These observations call for development of new therapeutic regimens for the HGBL-DHL entity.

Both MYC and BCL2 rearrangements are the defining characteristics of HGBL-DHL, which separately result in increased MYC and BCL2 proteins, thus providing particular opportunities for targeted therapies. BCL2 functions its pro-survival or antiapoptotic activity mainly through binding to BH3 domain-only proapoptotic factors and interfering with their proapoptotic capabilities, consequently beneficial for cell survival (7, 8). Evading apoptosis by upregulation of BCL2 contributes to tumor initiation and maintenance (9, 10), thereby targeting BCL2 is currently becoming an attractive therapeutic approach for the treatment of various malignant disorders. Recently, a couple of BCL2 inhibitors, the so-called “BH3 mimetics”, are massively developed (11). One of the most successful examples is the development of venetoclax, a selective and orally bioavailable BCL2 antagonist, which has shown unprecedented clinical responses with acceptable toxicities in numerous tumors types (12–15). In particular, venetoclax has shown impressive antitumor efficacies and been approved by US food and drug administration to treat chronic lymphocytic leukemia (CLL), even including those with 17p^-^ or TP53 mutations who belong to the worst prognostic CLL subtype (16). This drug also has been evaluated in several non-Hodgkin’s lymphoma (NHL) subtypes with variable clinical outcomes (13). Intriguingly, CAVALLI trial revealed that venetoclax combined with the standard-of-care chemotherapy yielded a high clinical response in DLBCL patients with overexpressed BCL2 (17). However, single venetoclax treatment less likely maintains a persistent antitumor effectiveness and frequently gives rise to acquired resistance following its continuous administration (18–20). These findings suggest that BCL2 blockade with venetoclax should combine with other drugs to overcome resistance and prevent disease progression.

MYC, a global transcription factor, modulates transcription of many genes that are involved in substantial different cellular processes, including cell proliferation and survival, and cell metabolism (21, 22). MYC overexpression is a hallmark of the aggressivity of malignancies, whereas MYC is widely considered intractable and undruggable (23). To circumvent this dilemma, a series of alternative therapeutic avenues are emerging to disrupt the MYC signaling and subsequently to antagonize its oncogenic activity (24, 25). The most well-known approach is to suppress the role of BRD4, a member of the bromodomain and extraterminal (BET) family. BET inhibitors lead to downregulation of MYC and its targets and exert the antitumor effect across a wide range of tumor types (26, 27). Importantly, BET inhibitor could cooperate with venetoclax to kill HGBL-DHL in preclinical models (28). Despite their considerable preclinical responses, most of BET inhibitors are unsuitable for clinical application due to very short half-life or unsatisfactory clinical efficacy. In this regard, compounds with differential mechanisms of action should become the research priority for enhancing the antitumor efficacy of venetoclax in the field of HGBL-DHL.

China-developed CS2164 (Chiauranib) is a novel orally active multitarget inhibitor, targeting three tumorigenesis-associated pathways that consist of angiogenesis related kinases (VEGFR1, VEGFR2, VEGFR3, and c-Kit), the chronic inflammation-related kinase CSF-1R, and the mitosis-related kinase Aurora B. This compound has shown broad preclinical antitumoral activities against several distinct human cancers (29–32). Notably, CS2164 is being evaluated in numerous clinical studies with promising clinical anticancer effects and tolerable toxicities (33). Our previous study suggested a potential role of CS2164 for the treatment of NHLs via perturbation of multiple signaling cascades. Particularly, CS2164 showed superior antilymphoma activity against MYC-arranged Burkitt lymphoma than other types of lymphoma, suggesting this agent might have a cytotoxic effect on other MYC-altered malignancies, containing HGBL-DHL (32). In the present work, we aimed to evaluate the combinatorial activity of CS2164 and venetoclax in in vitro and in vivo models of HGBL-DHL and to identify the main molecular mechanisms of action underlying the cooperative antitumor effects of the two drugs.

## Materials and Methods

### Cell Lines and Reagents

Three HGBL-DHL cell lines, including OCI-LY19, MCA and TMD8 which were kind gifts from MD Anderson Cancer Center Laboratory (Dr. Pham; ref (34–36) were cultured in RPMI-1640 (Hyclone, Thermo Scientific, Logan, UT, USA) supplemented with 10% fetal bovine serum (Gibco, Thermo Scientific, Grand Island, NY, USA) and 100 units/ml penicillin and 100 μg/ml streptoMYCin. All cell lines were tested and authenticated by an AmpFlSTR Identifiler PCR Amplification Kit (Thermofisher Scientific, USA) in our laboratory and were monthly tested for mycoplasma using PCR method. Venetoclax was purchased from Selleck Chemicals (Houston, Texas, USA). CS2164 (Chiauranib) was kindly donated by Shenzhen Chipscreen Biosciences (Shenzhen, China).

### Cell Viability Assay

Cell viability analysis, also known-as cell proliferation assay, was determined by Cell Counting Kit-8 (CCK-8) assay (Dojindo, Kumamoto, Japan). Briefly, HGBL-DHL cells were plated at 2 × 10^4^ cells per well in 96-well plates for 24 or 48 h. CCK-8 reagents (10 ul/well) were then added and incubated for additional 2 h, after which the absorbance at 450 nm was detected by a microplate reader (ELx800; BioTek Instruments Inc., Winooski, VT, USA). The inhibitory rate was calculated using the following formula: inhibition rate (%) = [1 − (absorbance of experimental group − absorbance of blank well)/(absorbance of control group- absorbance of blank well)] × 100%. The IC50 value (half maximal inhibitory concentration) of each cell line was calculated using GraphPad Prism 5.0. The assay was performed three times in triplicates, and the data presented as mean ± SD.

Based on the IC50 values, a panel of designated concentrations of CS2164 and venetoclax for each cell line was selected for the following cell viability assay to determine the synergism of the two drugs. The combination index (CI) was calculated using Compusyn software (ComboSyn Inc., Paramus, NJ, USA) according to the Chou-Talalay method, with definition for additive effect (CI = 1), synergism (CI < 1), and antagonism (CI > 1).

### Apoptosis Analysis

HGBL-DHL cells cultured in 24-well plates were treated with CS2164 and venetoclax either alone or in combination for 24 or 48 h. Cells were centrifuged and processed for Annexin V/PI Staining Kit (eBioscience, San Diego, California, USA) following the manufacturer’s instruction, and detected using a flow cytometry (BD Bioscience, Oxford, UK). We conducted three independent experiments to determine the apoptosis, and the data indicated as mean ± SD.

### Analysis of Mitochondrial Membrane Potential (Δψm)

The selected MCA and OCI-LY19 cells growing in 24-well plates were exposed to drugs for 24 h. Mitochondrial membrane potential was analyzed using a JC-1 kit (Signalway Antibody, Pearland, TX, US) as described by the manufacturer. Three experiments were performed to assess the level of mitochondrial membrane potential.

### Western Blot Analysis

Whole-cell extracts were electrophoresed on 8 to 12% gels and then transferred to PVDF membrane (Millipore, Billerica, MA, USA). The membranes were probed with specific primary antibodies and HRP-conjugated secondary antibodies (Abcam, Cambridge, UK). Proteins were visualized using ECL Western Blotting Detection Kit (Gene-Flow, Staffordshire, UK). The primary antibodies against AKT (CA4691S), p-AKT (CA4060S), BAX (CA5023S), BCL2 (CA4223S), BCL-XL (CA2762S), MYC (CA5605S), MCL1 (CA2538S), mTOR (CA2983S), p-mTOR (CA5536S), PUMA (CA3741S), PTEN (CA9188S), p85 (CA4257S), p110 (CA4249S), and β-actin (CA4970S) were purchased from Cell Signaling Technology (Danvers, Massachusetts, USA).

### Tumor Xenograft Models

The BALB/C nude mice (5–6 weeks of age, female and non-fertile) were purchased from the Beijing HFK bioscience CO.LTD and housed under specifc-pathogen-free (SPF) conditions following the animal care guidelines. The animal study was approved by the Animal Welfare Committee of Xiamen University. After receiving 4 Gy of total body irradiation, mice were injected subcutaneously on the flanks with 5 × 10^6^ MCA cells. After 3 days, mice were randomly assigned to four groups (n = 5, each group) administrated for 2 consecutive weeks: i. control group (0.2% methyl cellulose/0.1% Tween 80 in PBS); ii. CS2164 group (40 ug/g/d, oral gavage); iii. venetoclax group (50 ug/g/d, oral gavage); iv. combination of CS2164 and venetoclax group. Mice body weights were measured every day. At the 14th day, mice were euthanized, and tumors were harvested and weighed. At the end of the experiment, the greatest longitudinal diameter was measured by caliper and defined as the length (L) and the greatest transverse diameter as the width (W). Tumor volume (V) based on caliper measurements was calculated using the formula: V = (L × W^2^)/2 (37, 38).

### Statistical Analysis

Data were represented as mean ± S.D. of at least three independent experiments. The statistical analyses were performed by utilizing IBM SPSS 20.0 and GraphPad Prism 5.0 software. T-test was used to analyze the P value shown in **Table 1**. The one-way ANOVA method followed by Tukey’s multiple comparisons test was adopted to assess the difference between distinct treatment groups in analysis of cell apoptosis and mitochondrial membrane potential. For *in vivo* studies, the statistical significances were also performed using one-way ANOVA. P values <0.05 were considered as statistically significant.

**Table 1:** IC50 values of venetoclax or CS2164 for HGBL-DHL cells.

Cell Lines	Agents	IC50 ± S.D (μmol/L)		P value
		24 h	48 h	
TMD8	venetoclax	0.086 ± 0.005	0.08 ± 0.006	0.6439
	CS2164	25.38 ± 2.58	9.84 ± 0.64	0.0252
OCI-LY19	venetoclax	0.063 ± 0.006	0.026 ± 0.002	0.0009
	CS2164	20.67 ± 3.51	5.96 ± 0.72	0.0098
MCA	venetoclax	0.0219 ± 0.001	0.015 ± 0.002	0.004
	CS2164	67.87 ± 16.2	21.54 ± 4.87	0.0042

## Results

### *In Vitro* Antilymphoma Effect of Either Venetoclax or CS2164 on HGBL-DHL Cell Lines

OCI-Ly19, MCA and TMD8 cell lines which are HGBL-DHL cells were used in this study. Exposing these HGBL-DHL cells to either CS2164 or venetoclax for 24 and 48 h, cell viability was then assessed with a CCK-8 assay. On the basis of cell viability, the specific IC50 value for each cell line was obtained and shown in **Table 1**. Single-drug venetoclax induced significant loss of cell viability in three tested HGBL-DHL cells in dose- and time-dependent manners (**Table 1**). Treatment with CS2164 for 24 h resulted in a dose-dependent loss of cell viability with less sensitive in MCA cells than in OCI-Ly19 and TMD8 cells. The antilymphoma activity of CS2164 alone was clearly enhanced with prolonged treatment periods, manifested by decreased IC50 values in 48 h exposure as compared with 24 h exposure (**Table 1**).

### CS2164 and Venetoclax Are Synergistic in HGBL-DH Cellular Models

**Figure 1:**
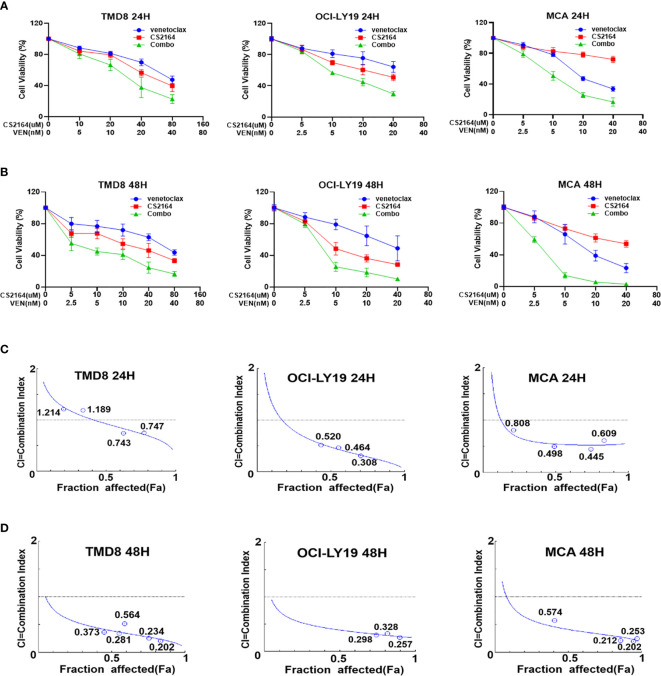
CS2164 synergizes with venetoclax to induce loss of cell viability in HGBL-DHL cells. HGBL-DHL cell lines (TMD8, OCI-LY19 and MCA) were separately treated with venetoclax and CS2164 alone or in combination for both 24 h **(A)** and 48 h **(B)**, and then assessed by CCK-8 assay to determine cell viability. CS2164 and venetoclax showed synergistic antilymphoma activity in HGBL-DHL cells at 24 h **(C)** or 48 h **(D)**, as indicated by combination index (CI) <1, which was calculated based on the Chou–Talalay method. Data were presented as mean ± S.D. of triplicates assays.

We next assessed the cytotoxic effects of combining CS2164 with venetoclax on the three HGBL-DHL cell lines. As shown in **Figure 1**, the antiproliferation activity of venetocalx was significantly potentiated in the presence of CS2164 in TMD8, OCI-LY19 and MCA cells, exhibiting dose-dependent and time-dependent manner (**Figures 1A, B**). We further calculated the combination index (CI), which is adopted to evaluate the drug-drug interaction with a quantitative definition for additive effect (CI = 1), synergism (CI < 1), and antagonism (CI > 1). Overall, strong synergism effects were observed in all examined HGBL-DHL cells as indicated by CI values < 1, irrespective of drug concentrations and treatment timepoints (**Figures 1C, D**).

**Figure 2:**
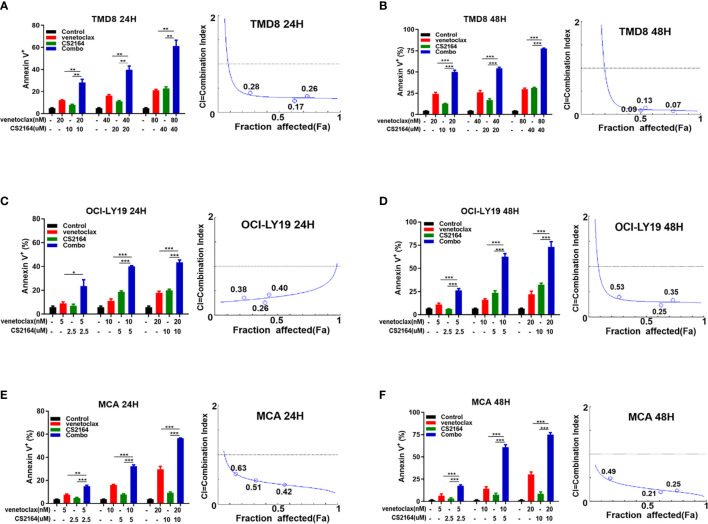
CS2164 and venetoclax cooperatively induce cell apoptosis of HGBL-DHL cells. Annexin *V/PI assay* was utilized to evaluate cell apoptosis of HGBL-DHL cells which were exposed to CS2164 and venetoclax alone or their combination for both 24 and 48 h*. CI values were calculated with* the Chou–Talalay method *based on apoptosis data. CI <1 indicates synergy, =* 1 addition, and <1 antagonism. CS2164 cooperated with venetoclax to promote TMD8 cell death at 24 **(A)** and 48 h **(B)**. The synergistic proapoptotic activity of CS2164 and venetoclax were also examined in OCI-LY19 **(C, D)** and MCA cells **(E, F)**. Data were presented as mean ± S.D. of triplicates assays. *P < 0.05, **P <0.01, ***P< 0.001.

To further verify the synergy of the combination of venetoclax and CS2164, apoptosis assays were performed using the Annexin V/PI dual staining in TMD8, OCI-LY19 and MCA cells. Building on the data of cell viability, three different concentrations of venetoclax (20, 40, and 80 nM for TMD8; 5, 10 and 20 nM for OCI-LY19 and MCA) and CS2164 (10, 20, and 40 uM for TMD8; 2.5, 5 and 10uM for OCI-LY19 and MCA) were selected as either single drug or their combination therapy for following apoptosis analyses. In TMD8 cells, treatment with venetoclax for 24 h slightly promoted apoptosis even in the high-dose group, and the proapoptotic ability of CS2164 was comparable to venetoclax alone. In marked contrast, CS2164 significantly enhanced the cell-killing efficacies of venetoclax when compared with each single drug (**Figure 2A**). Of importance, the synergistic proapoptotic effects of venetoclax/CS2164 combination was retained and even further strengthened with the extension of treatment to 48 h (**Figure 2B**). Combination of CS2164 plus venetoclax also demonstrated superior apoptosis-promoting activity in OCI-LY19 cell, regardless of treatment concentrations and time courses (**Figures 2C, D**). Similarly, CS2164 synergized with venetoclax to promote cell death in MCA cells at 24 and 48 h (**Figures 2E, F**). Taken together, CS2164 has a role to reinforce the antitumor potency of venetoclax in HGBL-DHL cells.

### The Synergy of CS2164 and Venetoclax in a HGBL-DHL Xenograft Murine Model

**Figure 3:**
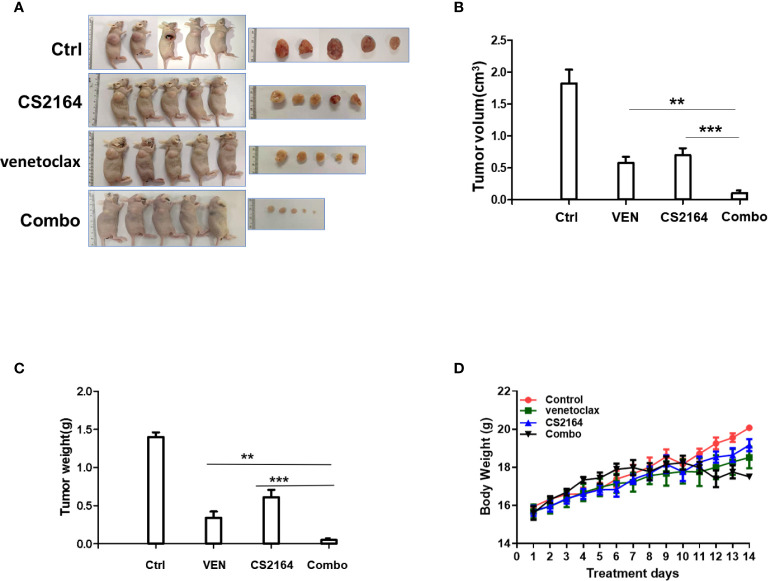
Synergistic antitumoral activity of CS2164 and venetoclax against HGBL-DHL-xenografted mice. MCA cells (5 × 10^6^) were engrafted subcutaneously into BALB/C nude mice. After 3 days of engraftment, HGBL-DHL-bearing mice were randomly allocated into four different treatment groups: control (Ctrl), CS1264, venetoclax, and the combination of CS2164 and venetoclax (Combo) for continuous 2 weeks therapy, respectively. **(A)** Images of tumor sizes were obtained at the end of the experiment. **(B)** Tumor volume and **(C)** tumor weight of each group were measured. **(D)** Mice body weight of each group was monitored daily during the entire treatment course. Data represents the mean ± SD. **P <0.01, ***P< 0.001.

Encouraged by these promising *in vitro* results indicated above, *in vivo* study was carried out to validate the antilymphoma activity of the combination regimen of CS2164 and venetoclax. Mice were engrafted with MCA cells and subsequently randomly assigned into four distinct groups: vehicle, CS2164, venetoclax, and the combination treatment groups for successive 14 days therapies. During the treatment course, body weight of mice was monitored daily to evaluate treatment-related side effects. At the end of the experiment, a total of 20 mice were euthanized and subcutaneous tumors were separately removed and photographed as well as weighed. In this study, either CS2164 or venetoclax alone modestly inhibited the *in vivo* cell growth of MCA, manifested by reduced tumor volumes. However, combination of CS2164 and venetoclax suppressed tumor growth more potent than both CS2164 and venetoclax monotherapy (**Figures 3A, B**). Accordingly, tumor weights were remarkably mitigated in the combination therapy by contrast to the other three groups (**Figure 3C**). Noticeably, no significant weight loss or other unacceptable toxicities were observed during the entire experiment course in the animals treated with CS2164 and venetoclax either alone or in combination (**Figure 3D**). Altogether, combination therapy of CS2164 and venetoclax is highly effective and safe for the management of HGBL-DHL *in vivo.*

### Combination of CS2164 and Venetoclax Induces DNA Damage and Impairs DNA Damage Repair in HGBL-DHL

**Figure 4:**
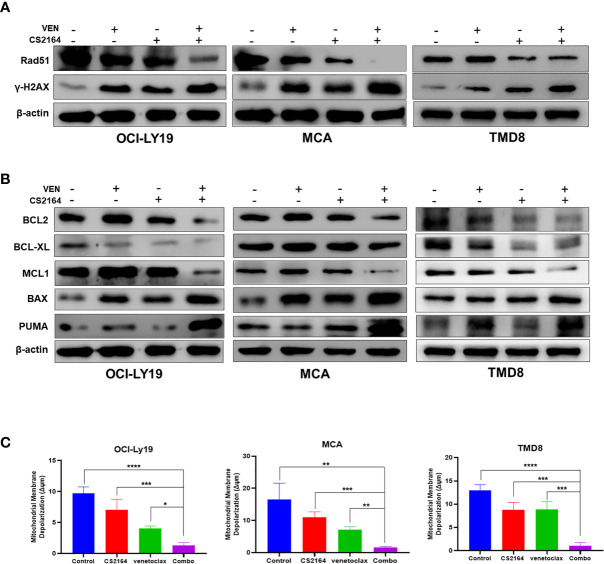
Combination of CS2164 and venetoclax impairs DNA repair capacity and disrupts the balance of BCL2 family members in HGBL-DHL cells. HGBL-DHL cells were treated with venetoclax (2.5 nM for OCI-LY19 and MCA; 10 nM for TMD8) and CS2164 (5 μM for OCI-LY19 and MCA; 20 μM for TMD8) as well as their combination for 24 h. **(A)** Western blot analysis of the expression of DNA damage biomarker γH2A.X and DNA repair protein Rad51. **(B)** BCL2 family proteins, including BCL2, BCL-XL, MCL, BAX, PUMA, were probed in all three tested HGBL-DHL cells. *β*-actin serves as loading controls. **(C)** The mitochondrial membrane potential (Δψm) was determined by a JC-1 assay in HGBL-DHL cells treated with CS2164 and venetoclax alone or their combination. Data were presented as mean ± S.D. of triplicates assays. *P <0.05, **P <0.01, ***P< 0.001.

Enhancement of DNA damage repair is an important feature accounting for the refractoriness of HGBL-DHL (39). We postulated that blockade of DNA damage repair might contribute to the synthetic lethality of CS2164 combined with venetoclax in HGBL-DHL cells. H2AX, a member of the histone H2A family, is phosphorylated at the very soonest in response of mammalian cells to DNA double-strand breaks (DSBs) and thus serves as a sensitive marker for detection of DNA damage (40). DSBs are usually repaired by two major repair pathways, homologous recombination repair and non-homologous end-joining, where the Rad51 recombinase plays a central role (41). We hereby examined the effects of the combination therapy on the level of phosphorylation of H2AX (designated γH2AX) and Rad51 by immunoblotting. As shown in **Figure 4A**, both CS2164 and venetoclax alone increased the abundance of γH2A.X but moderately influenced Rad51 expression in COI-LY19, MCA, and TMD8 cells (**Figure 4A**). Surprisingly, combination of both drugs not only dramatically elevated the level of γH2AX, but also markedly impaired the DNA repair executors Rad51 (**Figure 4A**). These results suggest that CS2164/venetoclax combination exerts a synergistic impact at least partially through disrupting the repair of DSBs and consequently results in an irreversible DNA damage in HGBL-DHL.

### CS2164 Enhances Venetoclax Cytotoxicity Through Regulation of BCL2 Family Proteins and Induction of Mitochondria-Dependent Apoptosis

Continuous BCL2 blockade with venetoclax often leads to upregulation of other antiapoptotic members of BCL2 family proteins, including MCL1 and BCL-XL, which confer to venetoclax resistance (20, 28, 42). In accordance with these findings, our results found that venetoclax alone indeed upregulated the expression of MCL1, as well as enriched the abundance of BCL2 proteins in OCI-LY19 and MCA cells (**Figure 4B**, left and middle panels), but not in TMD8 cells (**Figure 4B**, right panel). In addition, BCL2 inhibition with venetoclax elevated the level of BCL-XL in MCA cells, but not OCI-LY19 and TMD8 cells. In the meantime, blocking BCL2 function led to increased BAX and PUMA expression in all three tested HGBL-DHL cells. In the presence of CS2164, venetoclax dramatically decreased or abolished the expression of BCL2, MCL1 and BCL-XL in MCA, OCI-LY19, and TMD8 cells. More importantly, the combination of CS2164 and venetoclax significantly enhanced the level of a proapoptotic executor BAX and a proapoptotic sensitizer PUMA (**Figure 4B**).

During the process of mitochondrial-mediated apoptosis, the executor BAX moves onto the mitochondrial outer membrane and subsequently oligomerizes to form channels. This phenomenon causes mitochondrial membrane depolarization and releases cytochrome c into the cytoplasm, finally initiating caspase-mediated apoptotic cascades (7, 8). Therefore, we further determined the effect of increased BAX caused by the CS2164/venetoclax combination on the mitochondrial membrane potential (MMP, ΔΨm). As expected, cotreatment of CS2164 and venetoclax reduced the level of MMP more robust than CS2164, venetoclax, or the untreated groups in OCI-LY19, MCA and TMD8 cells (**Figure 4C**). Overall, the combination regimen disrupts the balance of the BCL2 family members, resulting in the accumulation of BAX and then triggering the mitochondrial apoptotic pathway in HGBL-DHL cells.

### Coexposure of CS2164 and Venetoclax Dramatically Downregulates MYC Expression and Attenuates the Activity of the PI3K/AKT/mTOR Signaling Pathway

**Figure 5:**
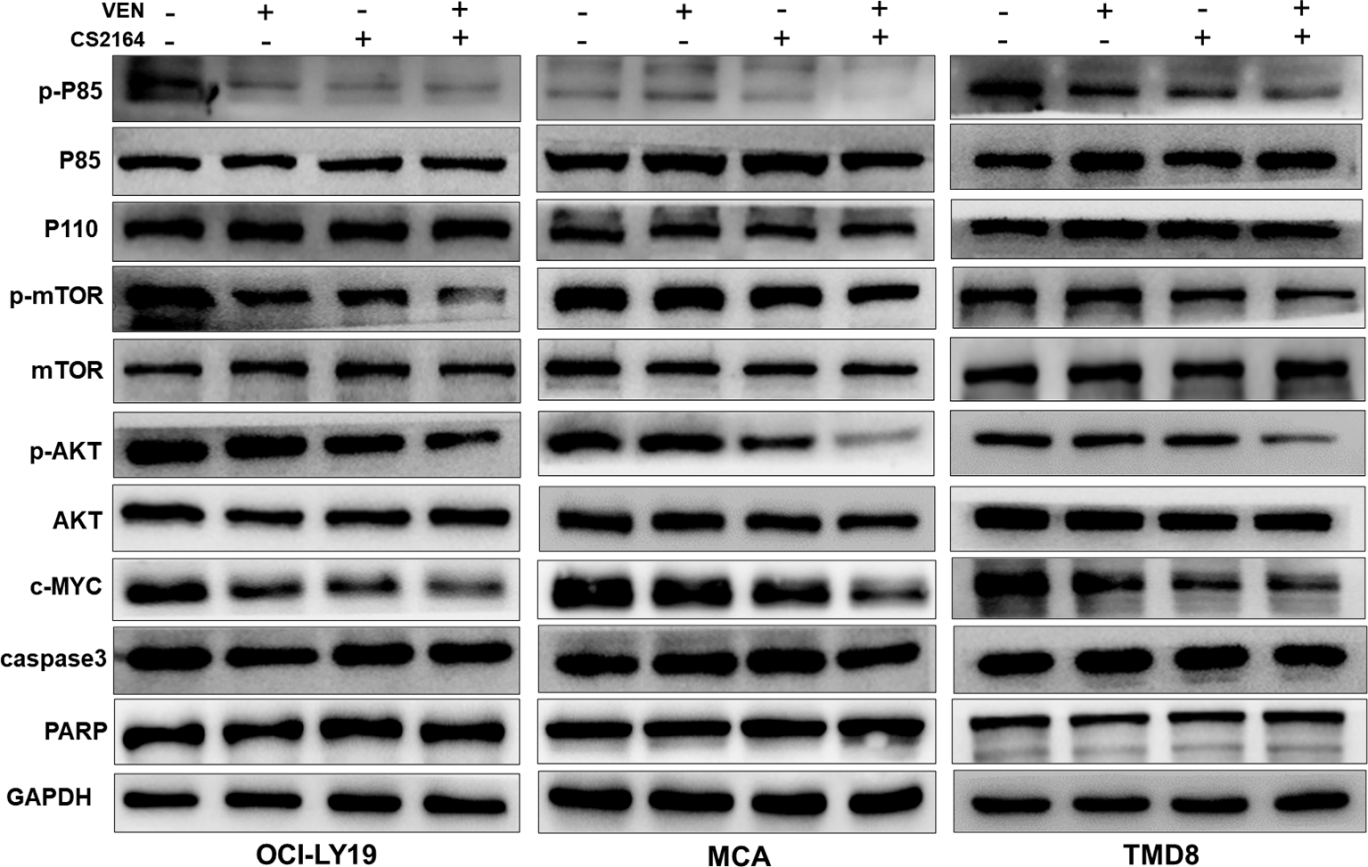
Cotreatment with venetoclax and CS2164 blunts the role of the PI3K pathway and inhibits the expression of MYC. HGBL-DHL cells were treated with venetoclax (2.5 nM for OCI-LY19 and MCA; 10 nM for TMD8), CS2164 (5 μM for OCI-LY19 and MCA; 20 μM for TMD8), or combination of both drugs for 6 h. Western blot analysis of the protein level of MYC and the PI3K signaling pathway components which consist of p85, p110, AKT, p-ATK, mTOR, p-mTOR and PTEN. Two apoptotic indicators, caspase-3 and PARP, were also detected in the same experimental context. *β*-actin acts as a loading control.

Finally, we investigated whether the combination treatment could modulate the expression of MYC protein in HGBL-DHL cell lines. Treatment with either CS2164 or venetoclax led to a moderate decrease of MYC expression in MCA, OCI-LY19, and TMD8 cells (**Figure 5**). Of note, CS2164 and venetoclax combination significantly diminished the expression of MYC protein, suggesting that downregulation of MYC is a potential molecular target for the synergy of CS2164 and venetoclax.

Dysregulation of phosphatidylinositol-3 kinase (PI3K) signaling is a common feature of B-cell neoplasms and contributes to MYC-driven lymphomagenesis (43, 44). Moreover, PI3K signaling can directly regulate MYC expression (45). Hence, PI3K signaling cascades, including p85, p110, AKT/p-AKT, and mTOR/p-mTOR, were examined in HGBL-DHL cells treated with venetoclax and CS2164 alone or in combination for 6 h. As displayed in **Figure 5**, either CS2164 or venetoclax individually had minimal impact on the PI3K pathway or even slightly activated some components of this pathway. However, CS2164 cooperated with venetoclax to markedly downregulate the phosphorylation level of P85, AKT, and mTOR. In the same treatment settings, neither single agents nor their combination induced changes of caspase 3 and PARP, indicating that the alteration of PI3K signaling has no correlation with cell death. Collectively, our data indicate that downregulation of MYC by CS2164 and venetoclax combination might be associated with inhibition of PI3K/AKT/mTOR signaling pathway.

## Discussion

HGBL-DHL is a rare and aggressive B-cell lymphoma with very poor survival and limited effective therapeutics (1, 2), informing that developing additional therapeutic approaches represent a crucial unmet need for patients with this form of lymphomas. In this study, we provided encouraging *in vitro* and *in vivo* evidence demonstrating that BCL2 blockade with venetoclax cooperated with CS2164, a potent antitumoral kinase inhibitor, against preclinical HGBL-DHL models. This cooperation seemed to be more effective on MCA cells than OCI-LY19 and TMD8 cells, potentially attributable to the different expression levels of BCL2 protein (46). *MYC* and *BCL2* rearrangements, the two hallmarks of HGBL-DHL, separately resulted in increased MYC and BCL2 proteins. As compared with monotherapies, combination of CS2164 and venetoclax remarkably downregulated the levels of *MYC* and *BCL2* proteins, suggesting that targeting the defining features of HGBL-DHL is a potential mechanism of action for the synergism. Induction of DNA damage response and perturbation of DNA damage repair contributed at least in part to the synergistic anti-lymphoma effect of CS2164 and venetoclax on HGBL-DHL. Enriching proapoptotic BCL2 family proteins and diminishing their antiapoptotic counterparts also played a necessary role for the synergy of CS2164 and venetoclax in HGBL-DHL cellular models. Additionally, exposure HGBL-DHL cells to the designated drug combination led to attenuation of the activity of PI3K/AKT pathway signaling, which are constitutively activated in B cell neoplastic disorders (43, 44).

*BCL2* rearrangement is a driver genetic lesion of HGBL-DHL and leads to enhanced BCL2 proteins, a critical antiapoptotic component of BCL2 family, which often confers to chemoresistance but might benefit from pharmacological BCL2 inhibition (1–6, 17). In fact, numerous preliminary clinical studies have demonstrated that venetoclax, an oral potent BCL2 antagonist approved to treat multiple hematological malignancies, plus standard-of-care regimens yield a survival benefit for BCL2-overexpressed DLBCL patients (17). Other preclinical reports indicated that venetoclax is able to promote HGBL-DHL cell deaths (28, 37, 47). In concert with previous published results, our data verified that venetoclax effectively killed HGBL-DHL cells *in vitro* and moderately ameliorated tumor burden in a xenograft mice model. Resistance to targeted drugs including venetoclax is a common feature during or after the planned treatment courses (48). Venetoclax resistance has extensively reported and is frequently associated with disruption of the balance of BCL2 family members. Herein, we observed that BCL2 inhibition with venetoclax upregulated its two major resistant determinants, MCL1 and BCL-XL, calling for combining with other compounds with different mechanisms of action to prevent or circumvent acquired resistance.

Co-modulation of BCL2 and MYC exhibits synergistic interactions against HGBL-DHL preclinical models, but most of these combined regimens fail to move forward to clinical evaluations (28, 49). This phenomenon is partially attributable to the “undruggable” characteristics of MYC and the poor bioavailability of MYC-modulating drugs (21–25). CS2164 was originally designed to function its cytotoxic effects mainly through interfering with tumor angiogenesis, mitosis as well as chronic inflammation (29). Preclinical and clinical studies have uncovered that monotherapy with CS2164 exerts broad antitumor potencies with favorable safety profiles in numerous solid and hematological malignancies (30–33). Our previous work demonstrated that the multi-kinase inhibitor CS2164 exhibits robust antilymphoma efficacies in a panel of aggressive B-cell lymphomas. Of great interest, *MYC*-translocated Burkitt lymphomas showed more sensitive to CS2164 treatment than DLBCL and mantel cell lymphomas (32), prompting us to investigate the effect of CS2164 alone or in combination with the BCL2 inhibitor venetoclax on the *MYC-*translocated HGBL-DHL. As a consequence, our study indicated that single CS2164 therapy effectively suppressed cell proliferation of HGBL-DHL cells in a dose- and time-dependent manner. Treatment with CS2164 greatly reduced the expression levels of MYC protein, implying that MYC might be a potential target for the antitumoral effectiveness of CS2164 in MYC-altered lymphomas. Notably, CS2164 and venetoclax were synergistic to lower HGBL-DHL cell viability and trigger mitochondrial mediated apoptosis. The synergy of CS2164 and venetoclax was linked to cooperatively decrease the abundance of BCL2 and MYC, two hallmark proteins of HGBL-DHL. In mice bearing HGBL-DHL cells, CS2164 combined with venetoclax showed a strong combinational antitumoral activity with acceptable toxicities, warranting further clinical investigation for the combination of CS2164 and venetoclax in HGBL-DHL patients.

Inducing DNA damage is considered an underlying molecular basis for the antitumor activity of venetoclax against various subtypes of malignant diseases, encompassing lymphomas (46, 50, 51). Venetoclax synergizes with many other agents, including multiple DNA damaging drugs, to induce DNA double-strand break (DSB) and to impair the capacity of DNA repair (51). Accordingly, we found venetoclax alone strikingly triggered DNA damage, evidenced by increasing the level of γH2AX foci, a well-featured DSB indicator, in HGBL-DHL. Treatment HGBL-DHL cells with venetoclax minimally weakened the process of Rad51-meidiated DNA damage repair. However, introduction of CS2164 to venetoclax significantly upregulated the abundance of γH2AX protein and remarkably blunted DNA repair mechanism, suggesting that modulation of DNA damage/repair process is a possible contributor for the synergism of CS2164 and venetoclax in HGBL-DHL.

Components of PI3K signaling pathway are frequently dysregulated in various tumors, particularly B cell originated neoplasms (52). Constitutive activation of the PI3K/AKT pathway could lead to MYC upregulation, while genomic or proteomic alterations of MYC are associated with resistance to PI3K pathway inhibitors in cancers (43–45). These observations provide a strong rationale for co-inhibition of PI3K/AKT pathway and MYC in cancer treatment. In the present study, beyond inhibiting MYC expression, CS2164 and venetoclax combination significantly attenuated the phosphorylation level of P85, mTOR and AKT, three important members of PI3K/AKT signaling cascade. These results supported that inactivation of this specific signaling cascade partially contributed to the synergistic antitumor effect of CS2164 and venetoclax on HGBL-DHL.

Previous studies have documented that activated PI3K/AKT signaling disrupts the balance of BCL2 family members at least in part by inactivating proapoptotic BAD protein and by upregulating antiapoptotic component MCL1, consequently beneficial for promoting cell survival and growth (53–55). Enhanced MCL1 and other BCL2 antiapoptotic members, such BCL-XL and BFL1, often contribute to resistance to BCL2 inhibition. In the other hand, persistent BCL2 blockade induces elevation of MCL1 and BCL-XL, thereby conferring to acquired resistance to BCL2 antagonists including venetoclax (20, 42). These studies illustrated that targeting these resistant contributors is an attractive therapeutic approach to improve the antitumoral activity of BCL2 inhibitors. Here, we demonstrated that CS2164 overcame the resistant mechanism of venetoclax through downregulating the level of MCL1 and BCL-XL as well as suppressing the expression of BCL2 in HGBL-DHL cellular models. Nevertheless, treatment with CS2164 alone had minimal effects on these BCL2 family proteins. Antagonizing pro-survival components by the CS2164 and venetoclax combination led to accumulation of proapoptotic executor BAX and proapoptotic sensitizer PUMA, followed by depolarizing the mitochondrial potential and initiating the intrinsic apoptotic signaling. These findings suggest that the combinatorial strategy with CS2164 and venetoclax could potentially overcome venetoclax resistance, a critical clinical dilemma, in the setting of HGBL-DHL models, further supporting the clinical evaluation of this combined regimen.

This study offers a promising rational treatment paradigm by combining CS2164 with venetoclax to strip HGBL-DHL of the protection from *MYC* and *BCL2* rearrangements. Cotreatment with CS2164 and venetoclax exhibited potent synergistic antitumoral effects primarily through diminishing the activity of the PI3K/AKT/mTOR pathway and preventing DNA repair. Of note, the synergy of CS2164 and venetoclax was closely associated with downregulation of the abundance of MYC and BCL2, *two defining proteins of HGBL-DHL.* Additionally, CS2164 cooperated with venetoclax to downregulate the level of BCL-XL and MCL1, two well-known factors contributing to venetoclax resistance, and to upregulate pro-apoptotic BAX and PUMA protein expression, thus tilting the BCL2 family balance to pro-death signaling cascade. Overall, the present study might potentially expand the clinical indications of CS2164 as single agent or in combination with venetoclax for the treatment of patients with HGBL-DHL disease.

## Data Availability Statement

The original contributions presented in the study are included in the article/supplementary material. Further inquiries can be directed to the corresponding authors.

## Ethics Statement

The animal study was reviewed and approved by the Animal Care and Use Committee of the First Affiliated Hospital of Xiamen University.

## Author Contributions

Conception and design: DY, MD, ZF, BX. Development of methodology: DY, GL, MD. Analysis and interpretation of data: DY, GL, LY, YJ, YS, QC, XM, LP, KY, MD. Technical support: DY, GL, MD, ZF, BX. Writing, review, and/or revision of the manuscript: LP, KY, MD, ZF, BX. Study supervision: ZF, BX. All authors contributed to the article and approved the submitted version.

## Funding

This work was financially supported by the National Natural Science Foundation of China (81770126, 81800196, 81770161, and 81800163), the Natural Science Foundation of Fujian Province (2018J01372, 2020J011246), Xiamen Municipal Bureau of Science and Technology (3502Z20184021) and Beijing Bethune Charitable Foundation (B19007CT).

## Conflict of Interest

LP was employed by Abbvie Company.

The remaining authors declare that the research was conducted in the absence of any commercial or financial relationships that could be construed as a potential conflict of interest.
